# Antimicrobial compounds from natural sources

**DOI:** 10.3389/fmicb.2013.00195

**Published:** 2013-07-15

**Authors:** Mirian A. Hayashi, Fernando C. Bizerra, Pedro Ismael Da Silva

**Affiliations:** ^1^Departamento de Farmacologia, Federal University of São Paulo - UNIFESPSão Paulo, Brazil; ^2^Departamento de Medicina, Federal University of São Paulo - UNIFESPSão Paulo, Brazil; ^3^Centro de Toxinologia Aplicada, Butantan InstituteSão Paulo, Brazil

Infectious diseases are one of the main causes of morbidity and mortality worldwide. Nowadays many infections are often caused by multi-resistant microorganisms resulting in difficult to treat diseases and, consequently, substantial increases in healthcare costs. The relative easy access to the antimicrobials and also the massive employment of these compounds for industrial purposes, including food production, have both strongly contributed to the progressive increase of resistant microorganisms. As a result, these multi-resistant microorganisms are reasserting themselves as worldwide threats.

Research into natural products has demonstrated significant progress in the discovery of new compounds with antimicrobial activity. In fact, nature is a generous source of compounds with the potential to treat diseases, including infectious diseases. Among the known sources of natural compounds with valuable antimicrobial activity, we highlighted the medicinal plants and marine and terrestrial organisms, including fungi and bacteria. Nevertheless, there is still a vast fauna and flora that once systematically explored, could provide additional antimicrobial leads and new drugs.

Thousands of natural products with the potential to act as antimicrobial compounds or as a structural lead compound still await further investigation.

In this Research Topic Ebook, we present several scientific studies mainly focused on natural products with antimicrobial activity, which are the case of the natural antimicrobial peptides (AMPs) and host defense peptides (HDPs). This topic also includes recent studies on the roles of honey hydrogen peroxide in antimicrobial activity against resistant microbial strains, as well as the use of essential oils for food preservation. Such a wide and interesting topic also gave us an opportunity to include diverse sources, including plants, terrestrial and sea animals. Not to mention the interesting and unusual sources such as coal or lignite, which may provide future antimicrobial compounds candidates. The recent development of a patented process to GMP standards (PA107470/GB), rendering the obtainment of carbohydrate derived fulvic acid (CHD-FA), stimulated Sherry et al. (2012) to study and describe for the first time a highly effective novel antiseptic effect of fulvic acid with exquisite biofilm activity that acts by disrupting cell membranes. The antifungic peptide from Amazonian Pink Toe spider Juruin, described by Ayroza et al. (2012) is another outstanding example of the potential contribution of a systematic exploration of nature aiming to provide additional antimicrobial leads and drugs.

In other words, nature is a generous source of compounds, with the potential to treat diseases, including infectious diseases. Studies exploiting the mechanism of action and the structure-activity aspects of these natural compounds may provide both additional antimicrobial leads and drugs, and also significant insight into potential possibilities to overcome the antimicrobial resistance.
